# Fecal samples and rectal swabs adequately reflect the human colonic luminal microbiota

**DOI:** 10.1080/19490976.2024.2416912

**Published:** 2024-10-22

**Authors:** Julia Rode, Linnea Brengesjö Johnson, Julia König, Ignacio Rangel, Lars Engstrand, Dirk Repsilber, Robert J Brummer

**Affiliations:** a School of Medical Sciences, Faculty of Medicine and Health, Nutrition-Gut-Brain Interactions Research Centre, Örebro University, Örebro, Sweden; b School of Health Sciences, Faculty of Medicine and Health, Örebro University, Örebro, Sweden; c Centre for Translational Microbiome Research, Dept Microbiology, Tumor and Cell Biology, Karolinska Institute, Solna, Sweden

**Keywords:** Gut microbiota, gut microbiome, intraluminal, feces, rectal, aspiration, sampling technique

## Abstract

The appropriateness of the fecal microbiota to adequately reflect the gut microbiota composition from more difficult to access luminal content at different colonic locations has been debated. Here, in a healthy population, luminal samples were collected from terminal ileum to rectum using an unique sampling technique without the need of prior bowel cleansing/preparation. Rectal swabs were collected immediately prior colonoscopy by an experienced physician, and fecal samples were collected at home by the participants themselves. Microbiota composition was evaluated as relative abundance, α-diversity and Bray–Curtis dissimilarities. Our data suggest that fecal samples and rectal swabs present noninvasive, easily accessible, low-cost sampling tools that are accurate proxies to characterize luminal large intestinal microbiota composition.

## Introduction

Human intestinal microbiota greatly impact host physiology and are involved in the pathophysiology of many chronic inflammatory, immunologic, and metabolic disorders (as reviewed in Afzaal et al. 2022).^1^ Studies on associations between intestinal microbiota and these disorders often rely on fecal microbiota, despite controversy whether fecal samples offer adequate reflection of the intestinal microbial ecosystem. Rectal swabs and fecal samples tend to be used interchangeably, providing highly reproducible microbiota profiles with similar α- and β-diversities.^2,3^ Both these sampling methods examine specimens from the distal part of the gastrointestinal tract and are often considered a proxy for the intraluminal microbiota of the colon. Yet, little is known about the microbiota composition at different locations along the healthy gut in its natural state, *i.e*., without previous preparation of the intestine. Hence, it is unclear whether fecal samples or rectal swabs adequately and uniformly represent the colonic microbiota. The few reported studies to date are equivocal. Most studies are based on samples collected during colonoscopy after bowel cleansing,^4–6^ which is known to impact the gut microbiota composition.^7^ Samples often originate from the distal colon,^4,8^ are based on biopsies^6,9^ or luminal brush samples^5^ representing mucosa-associated microbiota, and/or collected from individuals with gastrointestinal disorders.^4,5^ The microbiota of luminal aspirates obtained from ascending colon to rectum, after bowel cleansing and injection of distilled water, have been reported to resemble that of rectal swabs but not of fecal samples in subjects undergoing screening colonoscopy.^10^ Moreover, the relative abundance of microbial species in luminal aspirates collected during upper and lower endoscopy, after bowel cleansing, has been shown to differ between stomach, duodenum/jejunum and terminal ileum/caecum/descending colon, and all three differed significantly in UniFrac distances from fecal samples.^6^ In an unprepared bowel, fecal microbial composition and diversity were reported not to resemble that of luminal samples from stomach to ascending colon, collected by a pH-controlled capsule sampling device.^11^ Recently, sigmoidal luminal samples^8^ as well as rectal biopsy washes^12^ (collecting loosely adherent microbiota) obtained during unprepared distal colonoscopy from healthy subjects were shown to have similar α- and β-diversity measures as fecal samples.

## Design

**Figure 1.**
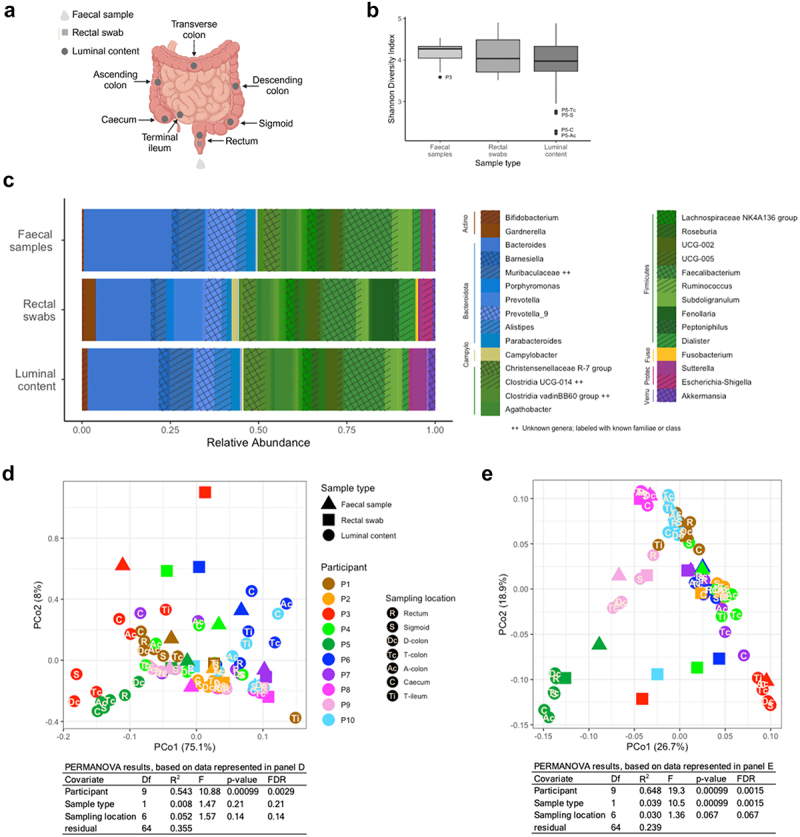
(a) overview of collected samples (type and sampling location); (b) α-diversity of fecal samples, rectal swabs, and luminal samples (averaged per participant) as Shannon diversity index. We tested for significant differences using a linear mixed effects model and Tukey’s *post hoc* test (with family-wise error rate (FWER) control), accounting for participants’ variability as a random effect. Significant differences are reported if adjusted *p* < 0.05. The central line represents the median, the box depicts 25^th^ to 75^th^ percentiles, and whiskers 1.5*IQR; (c) average composition as relative abundance of genera and phyla (colored at any abundance level, and depicted in legend if above 1% in all three sample types) of fecal samples, rectal swabs, and luminal samples; (d) PCoA of Bray–Curtis dissimilarities on level of phyla and (e) genera; see Supplementary Figure 1 for indication of the influence of sequencing depth and α-diversity. Samples are color-coded per participant, and symbols identify the different sample types, with location of luminal samples indicated. Summary of PERMANOVA results, with Benjamini–Hochberg’s false discovery rate (FDR), of Bray–Curtis dissimilarities of phyla and genera explaining influence of participant, sample type and sampling location on microbiota composition. Visual representation in panel a created with BioRender.com.

In our study, luminal samples were endoscopically collected from seven intestinal locations (rectum, sigmoid colon, descending colon, transverse colon, ascending colon, cecum, and whenever possible terminal ileum) based on anatomically defined landmarks without prior bowel cleansing (Figure 1a). We employed a contamination-avoiding aspiration technique during whole colonoscopy under conscious sedation. Rectal swabs were collected immediately prior to colonoscopy and fecal samples were collected within one week of colonoscopy. All samples were placed in DNA/RNA Shield^TM^ and stored frozen. Microbiota composition was assessed by 16S rRNA gene-based next-generation sequencing (v3-v4 region) and reads were further analyzed through DADA2, using SILVA v.138 as reference database.

P – participant; R – rectum; S – sigmoid colon; Dc, D-colon – descending colon; Tc, T-colon – transverse colon; Ac, A-colon – ascending colon; C – cecum; Ti, T-ileum – terminal ileum; Actino – Actinobacteriota; Campylo – Campylobacterota; Fuso – Fusobacteriota; Proteo – Proteobacteria; Verru – Verrucomicrobiota.

## Results

In a study population of 10 healthy adults (5 males/5 females, age 30 ± 5.6 y (22 to 43), BMI 23.8 ± 4.5 kg/m^2^), we found the composition of intraluminal samples from terminal ileum to rectum remarkably more similar than expected. Inter-individual differences dominated over comparatively small intra-individual differences across terminal ileal and colonic locations as well as sample types (*i.e*., luminal aspirates, rectal swabs, fecal samples), with minor location- and sample type-specific differences. Microbiota composition of fecal samples, rectal swabs, and luminal samples were remarkably similar in terms of relative abundance, with Firmicutes and Bacteroidota being the two predominant phyla (Figure 1c). Similarly, measures of α-diversity (denoted as Shannon Diversity Index, Figure 1b) did not significantly differ between those sample types. Bray–Curtis dissimilarities were dominated by inter-individual differences both on the taxonomic levels of phyla (Figure 1d) and genera (Figure 1e).

**Figure 2.**
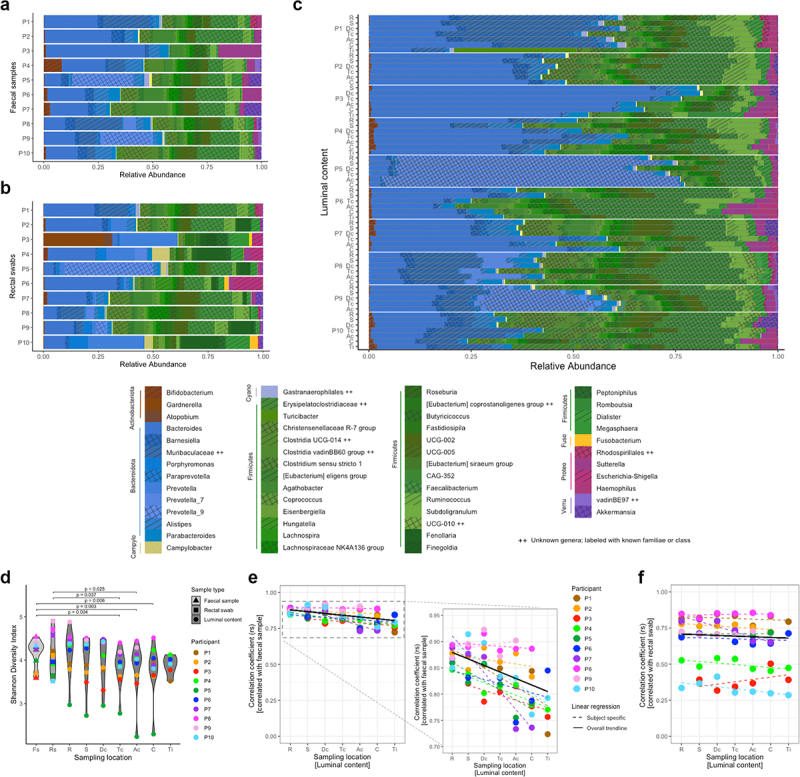
Detailed results for microbiota composition per participant, sample type, and sampling location. (a) relative abundances of genera and phyla (colored at any abundance level, and depicted in legend if above 2% in any sample) found in fecal samples, per participant, (b) rectal swabs, per participant, and (c) luminal samples, per participant and ordered by location within participants; (d) α-diversity of fecal samples, rectal swabs, and luminal samples at each intestinal location (from rectum to terminal ileum) as Shannon diversity index. Tested for significant differences by a linear mixed effects models and Dunnett’s *post hoc* test (with family-wise error rate (FWER) control) and reported if adjusted *p* < 0.05, including random effects to adjust for participants’ variability, where luminal samples per location were compared to fecal samples and rectal swabs, respectively. Samples are color-coded per participant, and symbols identify the different sample types, with location of luminal samples indicated; (e) Spearman correlations of relative abundances, on genus level, between fecal samples, and (f) rectal swabs with luminal samples at the various intestinal locations (from rectum to terminal ileum). Samples are color-coded per participant, with location of luminal samples indicated; all datapoints had Benjamini–Hochberg’s false discovery rate (FDR) < 0.05, dashed lines represent linear regression lines across all locations per participant and solid lines are based on median intercept and slope of all subject-specific linear regressions.

Given the predominance of inter-individual over intra-individual differences, compositions of individual fecal samples (Figure 2a), rectal swabs (Figure 2b), and luminal samples (Figure 2c) were further investigated. The relative abundance of microbiota ordered by location for each participant clearly indicated the intra-individual stability across sampling locations within the terminal ileum and colon. For comparison, the same data were depicted by location, emphasizing the inter-individual differences (Supplementary Figure 2). Average variance of the five topmost abundant genera (for list see Supplementary Table 1) of the two predominant phyla (Firmicutes and Bacteroidota), respectively, were mainly explained by participant (79.8 ± 7.5% and 80.0 ± 13.7%) and only minorly explained by sampling location (1.0 ± 0.9% and 2.8 ± 5.5%). Of those five topmost abundant genera of both phyla, only two were statistically different in abundance across sampling locations; *Christensenellaceae R-7 group* (phyla: Firmicutes) showed greater abundance in rectum (p = 0.043) and sigmoid colon (p = 0.036) compared to cecum, and *Prevotella_9* (phylum: Bacteroidota) showed lower abundance in sigmoid (p = 0.009) and descending colon (p = 0.037) compared to cecum. Furthermore, the relative abundances on the level of genera in luminal samples collected from rectum to terminal ileum showed a strong and significant correlation with those of fecal samples (Figure 2e) and rectal swabs (Figure 2f) for most subjects. Alpha diversity (denoted as Shannon Diversity Index) of fecal samples differed significantly from α-diversity of luminal samples collected from cecum, ascending and transverse colon (with a difference of 8.0%, 8.3% and 8.0%, and p = 0.006, 0.003 and 0.004, respectively). Alpha diversity of rectal swabs differed significantly from α-diversity of luminal samples collected from ascending and transverse colon (with a difference of 7.1% and 6.8%, and p = 0.025 and 0.037, respectively). In terms of α-diversity, neither fecal samples nor rectal swabs differed significantly from any of the other luminal sampling locations (rectum, sigmoid colon, descending colon, terminal ileum) (Figure 2d). This indicated a pattern with fecal samples and rectal swabs resembling the intraluminal microbiota the better the more distal in the colon.

P – participant; Fs – fecal samples; Rs – rectal swabs; R – rectum; S – sigmoid colon; Dc, D-colon – descending colon; Tc, T-colon – transverse colon; Ac, A-colon – ascending colon; C – cecum; Ti – terminal ileum; Campylo – Campylobacterota; Cyano – Cyanobacteria; Fuso – Fusobacteriota; Proteo – Proteobacteria; Verru – Verrucomicrobiota.

## Strengths and limitations

The sample size of this study is rather small, but ethically justified because of the highly invasive character of the investigational procedure. Yet, it gives valuable insights and lays the ground for larger studies, possibly in more heterogeneous or diseased study populations. Analyses approaches have been chosen to fit the small sample size and zero-inflated dataset. Additional analyses (data not shown) could validate the main conclusion of inter-individual differences outweighing intra-individual differences. Arguably, the availability of bioinformatics methods is broad, and others may be of interest for the analyses of this dataset in the future with a less generalized approach. Although all data were collected as close in time as possible, future studies should aim to standardize timing of fecal sample collection even better. Assessment of dietary data revealed that differences do not explain the inter-individual differences in microbiota composition (Supplementary Figure 3).

## Conclusion

Finally, the combined evidence suggests that fecal microbiota may not seem to be an appropriate surrogate marker for the entire gut microbiota from stomach to anus. Yet, our results support that fecal samples as well as rectal swabs adequately reflect the luminal microbiota composition of the human colon. Therefore, the choice of human microbial sample type depends on the respective research question.

## Materials and methods

### Study participants

In this cross-sectional study conducted from August 2018 to February 2019 at Örebro University Hospital (Örebro, Sweden), ten self-reported healthy subjects (aged 18–65 y) were recruited by public advertisement and included if none of the following exclusion criteria was met: known organic gastrointestinal disease (e.g. inflammatory bowel disease, irritable bowel syndrome, chronic diarrhea or constipation), history of or present gastrointestinal malignancy or polyposis, recent (gastrointestinal) infection (within last six months), history of major gastrointestinal surgery (e.g. gastric bypass), eosinophilic disorders of the gastrointestinal tract, current communicable disease (e.g. upper respiratory tract infection), malignant disease and/or patients who are receiving systemic anti-neoplastic agents, psychiatric diseases (e.g. dementia, depression, schizophrenia, autism, Asperger Syndrome) or other incapacity for adequate cooperation, chronic neurological/neurodegenerative diseases (e.g. Parkinson’s disease, multiple sclerosis), autoimmune disease and/or patients receiving immunosuppressive medications, major relevant allergies (e.g. food allergy, multiple allergies), chronic pain syndromes (e.g. fibromyalgia), chronic fatigue syndrome, obesity (body mass index >30 kg/m^2^) or metabolic syndrome, antimicrobial treatment or prophylaxis within the last three months, other chronic use of drugs that may affect the microbiome (e.g. proton pump inhibitors), females who are pregnant or breastfeeding, known clinically significant abnormal laboratory values, abuse of alcohol or drugs, probiotic intake within the last six weeks, bowel cleansing within the last six months, any clinically significant disease/condition which in the investigator’s opinion could interfere with the results of the trial.

Protocol deviations: Participants were included based on self-reported information at the time of screening. However, when present at the examination visit, one participant exceeded the upper limit for BMI and another participant reported undiagnosed depressive symptoms. All investigations were conducted as planned. Despite some variations, overall results of those subjects were not found to be extreme/outliers compared to the remaining participants, hence not excluded.

All subjects gave written informed consent before study start and were allowed to withdraw without stating a reason at any time. The study was conducted in accordance with the ethical principles set forth in the declaration of Helsinki and was approved by the Regional Ethical Review Board in Uppsala, Sweden (Dnr 2017/348) and is registered at ClinicalTrials.gov (NCT03918330). All participants were asked to keep a stable diet until sample collection was completed. Each participant was paid 2000 SEK (taxable income) for compensation of discomfort and time.

### Collection of fecal specimens

Subjects were asked to collect fecal samples stored in 9 mL DNA/RNA Shield^TM^ (Zymo Research, Sweden), a nucleic acid conserving reagent, at home from a natural bowel movement within a week of colonoscopy. Samples were typically collected in the morning (*i.e*. same day time as endoscopic sampling) 1–3 d prior to colonoscopy, with the exception of two individuals who collected their samples 6 or 7 d after colonoscopy. Collection from colonoscopy until 2 d after was omitted to avoid contaminations. Samples were placed into the home freezer immediately after collection and returned frozen in special cooling transporters (Sarstedt, Germany) to the research facility (max 2.5 d after collection) where they were stored at -80°C until analysis. Rectal swabs were taken three centimeters proximal of the anal verge by the gastroenterologist prior to the colonoscopy, ensured to not contain any fecal matter but merely mucus from the colorectal wall, and placed in 1 mL of DNA/RNA Shield^TM^ immediately.

Whole colonoscopy was performed after overnight fasting using a standard colonoscope (Olympus, Germany) with CO_2_ insufflation and without previous cleansing of the colon, but under conscious sedation using midazolam and alfentanyl. Luminal samples were collected as aspirates from seven defined locations. The order of sampling was rectum, sigmoid colon, descending colon, transverse colon, ascending colon, cecum, and terminal ileum. Sampling was performed starting with the most distal samples and continuing with sampling at increasingly proximal intestinal locations to avoid cross-contaminations. Luminal samples were collected using an innertube (LDPE Analytical Tubing, inner diameter 1.5 mm, outer diameter 3 mm; Reichelt Chemietechnik, Germany) which was placed in the working channel of the colonoscope. The tip of the tube was sealed by a small piece of cork to avoid contamination of the tube during placement in the working channel. A sterile guide wire (Dreamwire^TM^, Straight Tip; Boston Scientific, Sweden) was used to remove the cork once the tube was placed in the intended position. To sample luminal content, a vacuum was applied to the tube by a suction pump. The tubes containing luminal content were placed on ice immediately and transferred to DNA/RNA Shield^TM^ within five hours. All samples were stored frozen at -80°C until analysis. Due to difficulties during the procedure, not all samples of luminal content could be provided by all participants (see Supplementary Table 2 for an overview of all collected samples). As such, in several cases it was not possible to locate or pass the ileocecal valve for example due to substantial amounts of fecal matter or discomfort.

### Microbiota analysis

Fecal samples, rectal swabs, and luminal samples were analyzed for the composition of the microbiota by 16S rRNA gene-based next generation sequencing (NGS) in collaboration with the Centre for Translational Microbiome Research, Karolinska Institute and SciLifeLab in Stockholm, Sweden. Primers 341F and 805R were used to target the v3-v4 region of the 16S gene. Sequencing for 2 × 300bp using Illumina MiSeq was performed according to the protocol from Hugerth et al.^13^ In brief, this included Ion PGM OT2 400 Kit, Ion PGM Sequencing Kit 400, and Ion 318v2 chip (Gibco Life Technologies, USA), with analysis performed as per instructions of the manufacturer.

Reads from sequencing of the 83 samples were analyzed using the ampliseq automated pipeline by Nextflow (v.2.6.1)^14^ for trimming, filtering, and taxonomy assignment. Reads were truncated at forward and reverse positions 280 and 160, respectively, and mapped for amplicon sequence variants through DADA2^15^ with pooled sample processing, using SILVA (v.138)^16^ as taxonomy reference database. Samples with less than 3000 reads were excluded from downstream analysis (see Supplementary Table 2), resulting in 82 samples being used for subsequent analyses.

Count data were imported into R (v.4.3.2) for statistical analyses. Alpha diversity was calculated using the estimate.richness function in the phyloseq-package,^17^ based on raw ASV read counts. Statistical analysis of α-diversity was performed by mixed effects models, using functions lmer and glht from packages lme4^18^ and multcomp,^19^ respectively, accounting for participants’ paired data with random effects. Sample type comparisons were then assessed by Tukey’s *post hoc* test based on averages per participant for luminal content (as presented in Figure 1b). Sampling location comparisons (as presented in Figure 2d) were further investigated by Dunnett’s *post hoc* test based on available samples per participant. Relative differences in α-diversity were calculated based on the estimated fitted values of the mixed effects models, and reported in percentage. For further downstream analysis, read counts were normalized to relative abundance per sample, hence controlled for sequencing depth (a summary of sequencing depth data can be found in Supplementary Figure 4 and Supplementary Information S1). Package vegan^20^ was used for β-diversity analysis, where function adonis2 was utilized for PERMANOVA tests with Type III sums of squares analysis to assess impact of participant, sample type, and location on sample composition dissimilarities. Variance component analysis was performed on log-transformed abundance of the five topmost abundant Firmicutes and Bacteroidota, respectively, using function remlVCA in package VCA.^21^ Negative binomial mixed models, with participant as random effect, were performed on count data of the same five topmost genera, using packages glmmTMB^22^ and emmeans^23^ with pairwise comparisons using Tukey’s *post hoc* test. Spearman correlations were employed due to the non-parametric and zero-inflated nature of the taxonomic data, and computed based on the full composition of available samples, including zero entries for missing taxa when comparing fecal or rectal swab datasets with luminal data. Graphical displays were created using ggplot.^24^ All statistical measures were multiplicity-corrected, and false discovery rate based on Benjamini–Hochberg,^25^ as well as family-wise error rate incorporated in the mixed effects tests, are reported. Comparisons were considered statistically significant for adjusted *p* < 0.05.

### Assessment of dietary data

A 3 d (two typical weekdays, one typical weekend day) food diary was filed by each participant within approximately one week of colonoscopy. Average energy and nutrient intake was assessed using Dietist Net, and statistically analyzed in R.

## Supplementary Material

Supplemental Material

## Data Availability

The 16S rRNA gene sequencing reads are available on NCBI SRA under BioProject PRJNA1049424.

## Code availability

For further details of analyses parameters and R-code, see public repository on GitHub https://git.oru.se/ngbi/spim for custom code for data processing, statistical analysis and figure generation.
