# Supplementary material for: Fecal samples and rectal swabs adequately reflect the human colonic luminal microbiota
Source: Gut Microbes. 2024 Oct 22;16(1):2416912. doi: 10.1080/19490976.2024.2416912 (PMC11508938; doi:10.1080/19490976.2024.2416912)
Supplement: Supplemental Material [file KGMI_A_2416912_SM9483.docx]

**SUPPLEMENTARY MATERIALS**

Fecal samples and rectal swabs adequately reflect the human colonic luminal microbiota

Julia Rode^1,2,*^, Linnea Brengesjö Johnson^1^, Julia König^1^, Ignacio Rangel^1^, Lars Engstrand^3^, Dirk Repsilber^1^, Robert J Brummer^1,**^

**
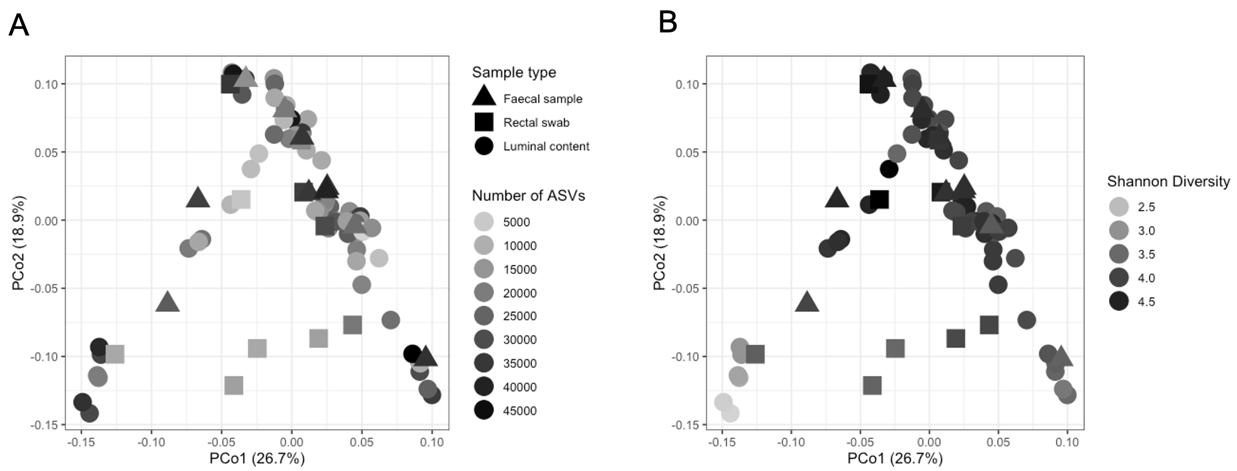
**

**Supplementary Figure 1:** PCoA Bray-Curtis dissimilarities on the level of genera with indication if **A** sequencing depth or **B** α-diversity may be hidden variables that influence variation in our data. The same data is presented in Figure 1E, but colored differently.


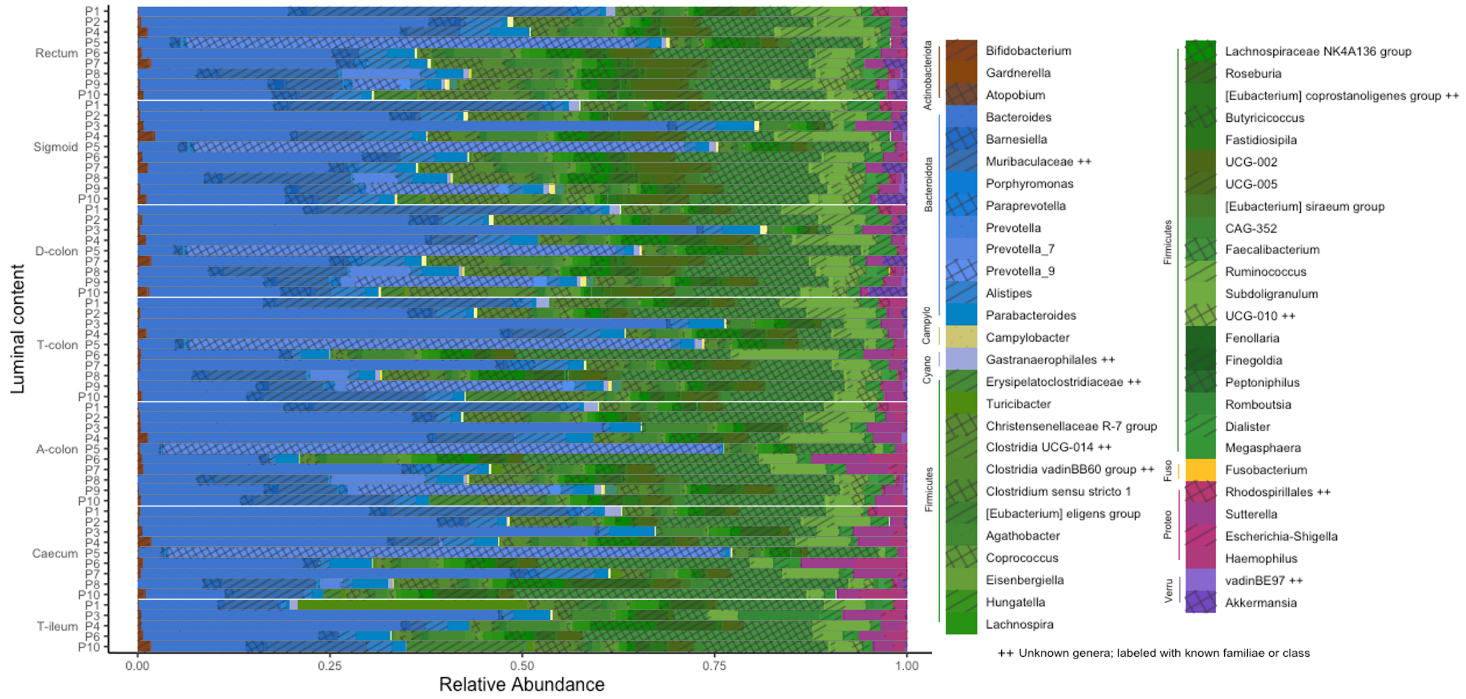


**Supplementary Figure 2:** Microbial composition as relative abundance of genera and phyla of luminal samples per intestinal location and ordered by participants within locations (colored at any abundance level, and depicted in legend if above 2% in any sample). The same data is presented in Figure 2C, but ordered differently.

P – participant; P – participant; D-colon – descending colon; T-colon – transverse colon; A-colon – ascending colon; T-ileum – terminal ileum.; Campylo – Campylobacterota; Cyano – Cyanobacteria; Fuso – Fusobacteriota; Proteo – Proteobacteria; Verru – Verrucomicrobiota.


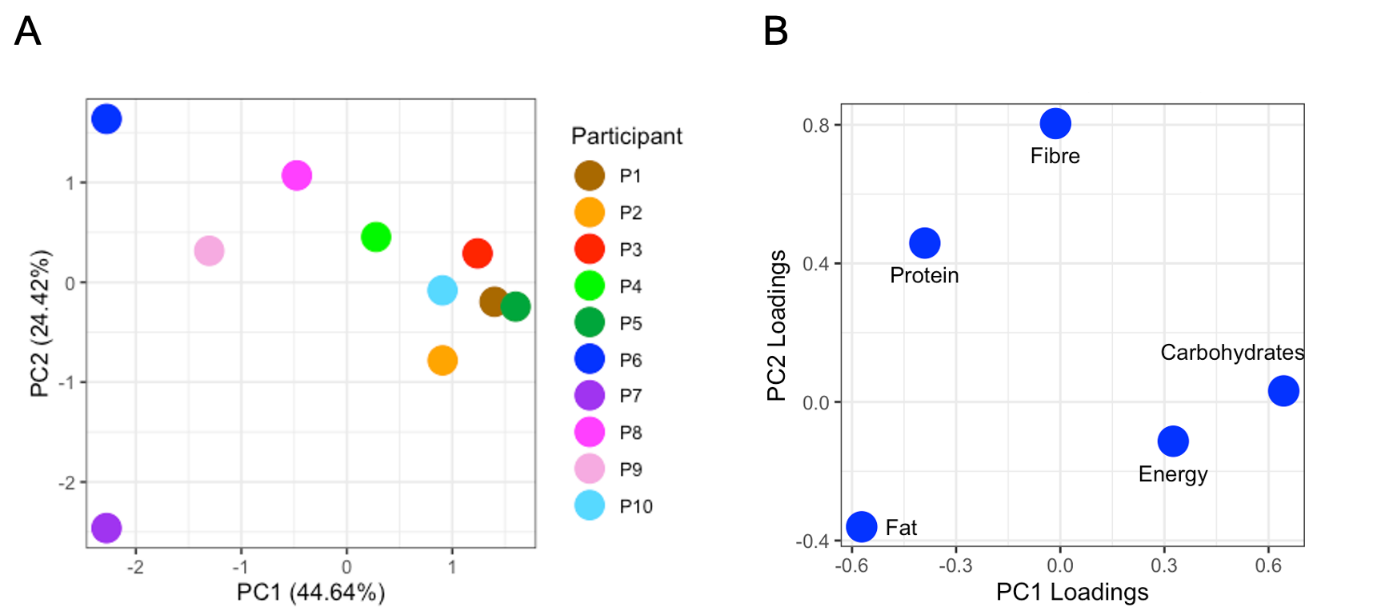


**Supplementary Figure 3:** Overview of dietary data (assessed by 3-day food diary) in terms of energy intake [kcal], intake of protein, fat and carbohydrates [all E%] and fiber intake [g/MJ] normalized to energy intake. **A** PCA scores plot; **B** PCA loadings plot visualizing the impact of each variable. Samples are color-coded per participant. P – participant.

**Supplementary Table 1:** Five topmost abundant genera for the two predominant phyla Firmicutes and Bacteroidota.

| **Family** | **Genus** | **Average relative abundance*** |
| --- | --- | --- |
| Top 5 of phylum Firmicutes | | |
| *Ruminococcaceae* | *Faecalibacterium* | 13.1% |
| *Christensenellaceae* | *Christensenellaceae R-7 group* | 3.5% |
| *Ruminococcaceae* | *Subdoligranulum* | 3.2% |
| *Lachnospiraceae* | *Agathobacter* | 2.5% |
| *Lachnospiraceae* | *Lachnospiraceae NK4A136 group* | 2.0% |
| Top 5 of phylum Bacteroidota | | |
| *Bacteroidaceae* | *Bacteroides* | 24.8% |
| *Prevotellaceae* | *Prevotella_9* | 7.8% |
| *Muribaculaceae* | Unknown | 7.0% |
| *Rikenellaceae* | *Alistipes* | 2.8% |
| *Tannerellacea* | *Parabacteroides* | 2.5% |

*Average relative abundance in all luminal content samples independent of location

**Supplementary Table 2:** Overview of samples collected and analyzed per participant, sample type and location.

| **Sample type Sampling location** | **P1** | **P2** | **P3** | **P4** | **P5** | **P6** | **P7** | **P8** | **P9** | **P10** | **n** |  |
| --- | --- | --- | --- | --- | --- | --- | --- | --- | --- | --- | --- | --- |
| Fecal sample | ✓ | ✓ | ✓ | ✓ | ✓ | ✓ | ✓ | ✓ | ✓ | ✓ | 10 |  |
| Rectal swab | ✓ | ✓ | ✓ | ✓ | ✓ | ✓ | ✓ | ✓ | ✓ | ✓ | 10 |  |
| Luminal content Rectum | ✓ | ✓ | ✘ * | ✓ | ✓ | ✓ | ✓ | ✓ | ✓ | ✓ | 9 |  |
| Luminal content Sigmoid | ✓ | ✓ | ✓ | ✓ | ✓ | ✓ | ✓ | ✓ | ✓ | ✓ | 10 |  |
| Luminal content Descending colon | ✓ | ✓ | ✓ | ✓ | ✓ | ✘ ** | ✓ | ✓ | ✓ | ✓ | 9 |  |
| Luminal content Transverse colon | ✓ | ✓ | ✓ | ✓ | ✓ | ✓ | ✓ | ✓ | ✓ | ✓ | 10 |  |
| Luminal content Ascending colon | ✓ | ✓ | ✓ | ✓ | ✓ | ✓ | ✓ | ✓ | ✓ | ✓ | 10 |  |
| Luminal content Caecum | ✓ | ✓ | ✓ | ✓ | ✓ | ✓ | ✓ | ✓ | ✘ * | ✓ | 9 |  |
| Luminal content Terminal ileum | ✓ | ✘ * | ✓ | ✓ | ✘ * | ✓ | ✘ * | ✘ * | ✘ * | ✓ | 5 |  |
| * sample was not collected from participant, ** sample was excluded from analysis due to very low number of reads. | | | | | | | | | | | | |

**Supplementary Information S1:** Assessment of sequencing depth

Statistical investigation of ASV counts as dependent on sample type (mixed model, random effect for participant, *post hoc* Tukey test), showed statistically significant differences comparing rectal swabs and fecal samples (p=0.016) as well as luminal content compared to fecal samples (p=0.009). ASV counts in rectal swabs compared to all luminal content were statistically non-significant, also when comparing to luminal content at each location separately. Similar analyses of fecal samples compared to luminal content of different locations showed statistically significant difference only for the sigmoid colon (p=0.045). ASV counts of luminal contents did not differ significantly between different sampling locations. See Supplementary Figure 4.

**
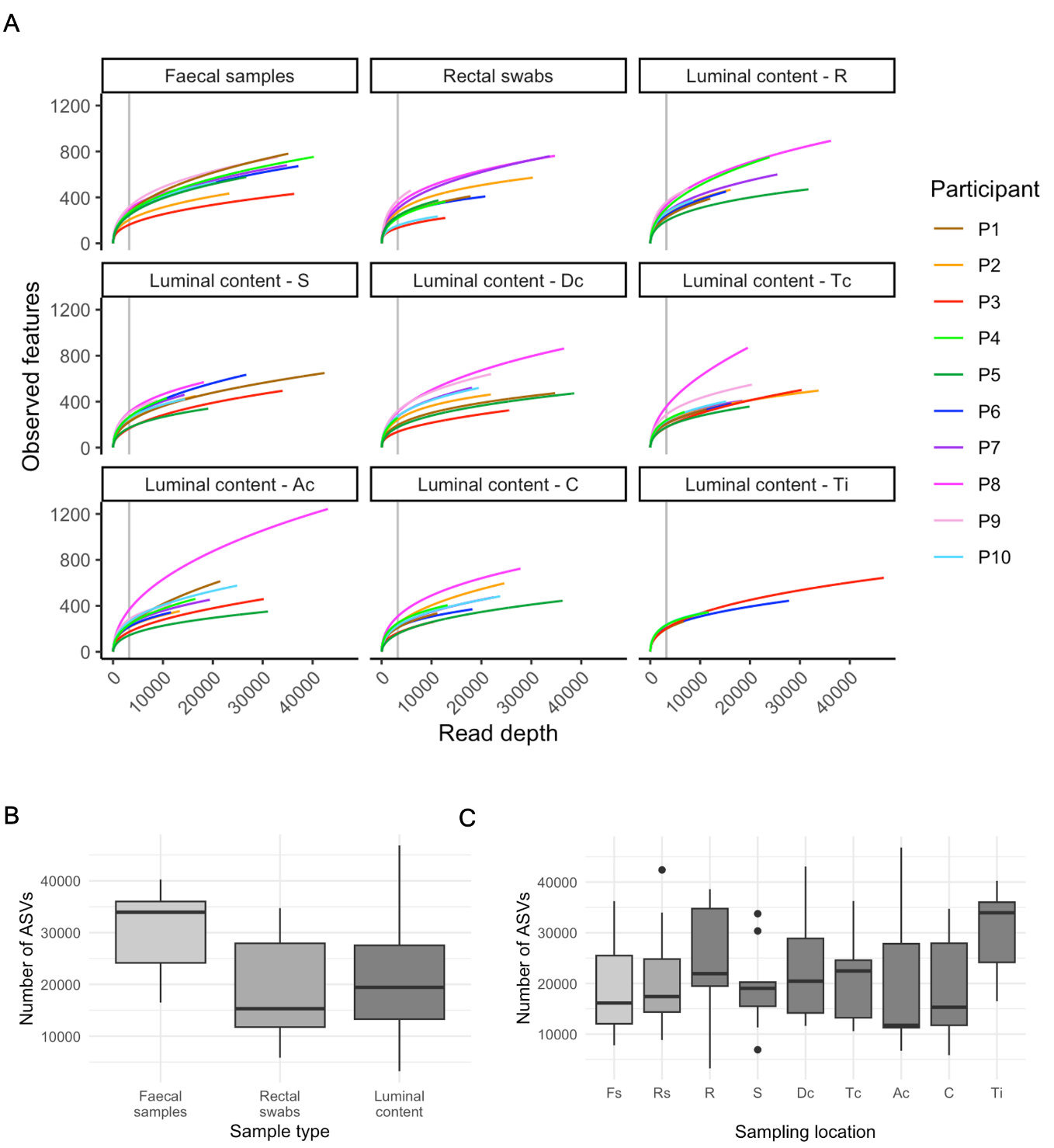
**

**Supplementary Figure 4:** Sequencing depth. **A** Alpha rarefaction curves for the number of observed features by read depth, depicted by sample type, sampling location, and subjects. Vertical line represents the lowest read depth in all included samples. **B** Number of ASVs found in each sample type. **C** Number of ASVs found in luminal samples at the different sampling locations. For **B** and **C** the central line represents the median, the box depicts 25^th^ to 75^th^ percentiles, and whiskers 1.5*interquartile range (IQR). P – participant; Fs – fecal samples; Rs – rectal swabs; R – rectum; S – sigmoid colon; Dc – descending colon; Tc – transverse colon; Ac – ascending colon; C – caecum; Ti – terminal ileum.
